# Fasting promotes acute hypoxic adaptation by suppressing mTOR-mediated pathways

**DOI:** 10.1038/s41419-021-04351-x

**Published:** 2021-11-03

**Authors:** Ruzhou Zhao, Xingcheng Zhao, Xiaobo Wang, Yanqi Liu, Jie Yang, Shuai Jiang, Xiang Zhou, Bo Jiao, Lin Zhang, Yong Liu, Zhibin Yu

**Affiliations:** 1 Department of Aerospace Physiology, Air Force Medical University, Xi’an, China; 2 Department of Nuclear Medicine, Xijing Hospital, Air Force Medical University, Xi’an, China

**Keywords:** Autophagy, Metabolism

## Abstract

Rapid adaptation to a hypoxic environment is an unanswered question that we are committed to exploring. At present, there is no suitable strategy to achieve rapid hypoxic adaptation. Here, we demonstrate that fasting preconditioning for 72 h reduces tissue injuries and maintains cardiac function, consequently significantly improving the survival rates of rats under extreme hypoxia, and this strategy can be used for rapid hypoxic adaptation. Mechanistically, fasting reduces blood glucose and further suppresses tissue mTOR activity. On the one hand, fasting-induced mTOR inhibition reduces unnecessary ATP consumption and increases ATP reserves under acute hypoxia as a result of decreased protein synthesis and lipogenesis; on the other hand, fasting-induced mTOR inhibition improves mitochondrial oxygen utilization efficiency to ensure ATP production under acute hypoxia, which is due to the significant decrease in ROS generation induced by enhanced mitophagy. Our findings highlight the important role of mTOR in acute hypoxic adaptation, and targeted regulation of mTOR could be a new strategy to improve acute hypoxic tolerance in the body.

## Introduction

With the growing need for emergency ascents to high altitudes (HA) within several hours, rapid adaptation to extreme HA is a challenge that has become particularly important in recent years [1–4]. Regretfully, the time-consuming acclimation regimens cannot meet these needs [2, 3]. No established rapid adaptation schemes have been reported to be effective, and the mechanism underlying acclimation or adaptation to HA remains unclear. Fortunately, for the first time, we show that fasting pretreatment for 72 h can significantly improve the adaptability to acute extreme hypoxia, suggesting that fasting can be an effective method for rapid adaptation to HA.

In this study, we used SD rats as an in vivo model. By simulating a hypobaric hypoxic environment, we identified fasting as an effective way to rapidly adapt to hypoxic environments and downregulate mTOR, which is a pivotal factor that contributes to the strengthened adaptability of SD rats to hypoxia. From the perspective of systemic energy expenditure and generation, the underlying mechanisms of mTOR are thoroughly explored in this article.

## Materials and methods

### Experimental animals

Adult male SD rats (10−12 weeks old, body weight > 300 g) were used in this study. All rats were raised in specific pathogen-free (SPF) cages with suitable temperature (25 ± 5 °C) and humidity (50 ± 5%). The fasting rats were deprived of standard rodent feed but were provided water ad libitum. All experiments involving animals were approved by the Ethics Committee for Animal Care and Use of Air Force Medical University.

SD rats were randomly assigned to four groups. Con (rats at baseline altitude, 412 m), F (fasting for 72 h at baseline altitude, 412 m), H (rats exposed to a simulated altitude of 7620 m within 24 h until death), FH (rats fasted for 72 h and then exposed to a simulated altitude of 7620 m for 24 h). All rats were anesthetized after treatment, and then fresh heart and liver tissues were collected and frozen at −80 °C or fixed with 4% paraformaldehyde (CWBIO, Beijing, China).

### Cardiomyocyte culture

The rat myocardial cell line H9C2 was cultured in Dulbecco’s modified Eagle’s medium (DMEM) supplemented with 10% fetal bovine serum (Procell, Wuhan, China) in 5% CO_2_ at 37 °C.

H9C2 cells were routinely cultured in normoglycemic (1 g/L glucose) DMEM before any treatments (Con group). To simulate fasting pretreatment, the cells were cultured in hypoglycemic (0.5 g/L glucose + 0.5 g/L mannitol) DMEM for 72 h (F group). For acute hypoxia treatment, cells were exposed to 1% O_2_ for 24 h (H group and FH group) using a tri-gas incubator (Heal Force, Shanghai, China).

For mTOR gene interference, the cells were transfected with mTOR-targeting siRNA (si-mTOR group). A scrambled siRNA was used as the negative control (si-NC group). Acute hypoxia exposure was the same as that described above (si-NC + H group and si-mTOR + H group).

### Hypobaric hypoxia simulation

Hypobaric hypoxia was simulated by our laboratory-developed hypobaric chamber system, which consists of a vacuum pump, a control panel, a pressure and flow control system, and hypobaric chambers. The temperature and humidity in the chambers were maintained at 25 ± 5 °C and 50 ± 5%. Hypobaric hypoxia could be mimicked via a vacuum pump after the pressure altitude was set with the control panel. The ascending and descending speeds were controlled at a rate of 10 m/s by the flow control system. Airflow of 1 L/min per rat was also sustained to ensure fresh air in the chambers.

### Hematoxylin−eosin staining

Heart and liver tissues were fixed with 4% paraformaldehyde for 24 h. After the paraffin sections were prepared, dewaxing, hematoxylin staining, eosin staining, and dehydration were sequentially performed. Images were captured using photomicroscopy (Olympus, Tokyo, Japan).

### Echocardiography

M-mode echocardiography was performed with a preclinical small animal ultrasound imaging system (Vinno Corp., Suzhou, China). For the rats without fasting, echocardiography was immediately conducted before ascending (Con) and after descending (H); for the rats that underwent fasting, echocardiography was immediately performed before fasting (Con), after fasting, which was before ascending (F), and after descending (FH).

The rats were anesthetized with 3% isoflurane and maintained with 1~1.5% isoflurane before the ultrasonography examination. The heart rate (HR), left ventricular internal diameter at end-systole (LVIDs), left ventricular internal diameter at end-diastole (LVIDd), left ventricular end-diastolic volume (LVEDV), and end systolic volume (LVESV) were recorded. Left ventricle ejection fraction (LVEF) and left ventricle fractional shortening (LVFS) were calculated as follows: LVEF% = [(LVEDV − LVESV)/(LVEDV) × 100] and LVFS% = [(LVIDd − LVIDs)/(LVIDd) × 100].

### Western blotting

Tissues or cardiomyocytes were dissociated by lysis reagent, and then protein was extracted. Protein was quantified using a BCA kit (Thermo Fisher Scientific, MA, USA). Western blotting was performed as previously described. Briefly, 20 μg of protein was electrophoresed and transferred to a polyvinylidene difluoride membrane (Millipore, Schwalbach, Germany). The membrane was blocked with 5% BSA in Tris-buffered saline with 0.1% Tween 20, followed by incubation with primary antibodies overnight at 4 °C. Then, the membrane was incubated with appropriate HRP-linked secondary antibodies for 1 h at room temperature. The membrane was finally imaged using an Odyssey scanner (LI-COR Biosciences, NE, USA) and analyzed using NIH ImageJ software. The antibodies used in this experiment are described in Supplementary Tables S1 and S2.

### Immunohistochemistry

FASN and ACC1 expression was analyzed by immunohistochemistry as previously described [5]. Tissue paraffin sections were incubated with 3% hydrogen peroxide (Servicebio, Wuhan, China) for 20 min to block endogenous peroxidase after dewaxing and antigen repair was performed. The sections were blocked with 3% BSA, followed by incubation with primary antibodies overnight at 4 °C. After the samples were incubated with HRP-conjugated secondary antibodies, diaminobenzidine solution (Millipore, Schwalbach, Germany) was used for color development.

### Immunofluorescence staining of tissue sections

For single labeling of SREBP1, frozen sections were prepared and fixed with 4% paraformaldehyde for 20 min. The frozen sections were subjected to antigen repair, blocked and incubated with primary antibodies overnight at 4 °C, followed by incubation with Alexa Fluor 594-conjugated anti-rabbit secondary antibodies. Then, 1 μg/mL Hoechst 33258 solution (Thermo Fisher Scientific, MA, USA) was used for nuclear staining.

For double labeling of LC3 and BNIP3, paraffin sections were prepared, and a TSA fluorescein kit (PerkinElmer, MA, USA) was used. Staining was performed according to the kit instructions. The first few steps were the same as those in the immunohistochemistry experiment. Then, the paraffin sections were incubated with primary antibodies (LC3) overnight at 4 °C and HRP-conjugated anti-rabbit secondary antibodies for 50 min at room temperature, followed by incubation with the TSA-FITC solution (PerkinElmer, MA, USA). Next, incubation with the second primary antibody (BNIP3) and HRP-conjugated anti-rabbit secondary antibody was conducted in a similar manner. TSA-CY3 solution (PerkinElmer, MA, USA) was used for the next step. Finally, DAPI was used for nuclear staining.

The stained sections were examined by Pannoramic MIDI and evaluated through Pannoramic Viewer (3DHISTECH, Budapest, Hungary). At least ten fields per tissue sample were randomly collected. NIH ImageJ software was used to analyze the fluorescence intensity and colocalization puncta.

### Oil Red O staining

Frozen liver sections were stained with Oil Red solution (Servicebio, Wuhan, China) for 8–10 min in the dark. The sections were immersed in 60% isopropanol for differentiation, followed by incubation with hematoxylin and bluing in tap water. The red-stained lipid droplets were observed via light microscopy. Quantification of the Oil Red O intensity (Oil Red O staining area per cell) was performed using ImagePro Plus software (Media Cybernetics, Georgia, USA).

### Transmission electron microscopy (TEM)

Fresh cardiac tissues were collected from the groups of rats, immediately fixed with 4% glutaraldehyde (Servicebio, Wuhan, China) and then trimmed into 0.1 × 0.1 × 0.1 cm^3^ blocks for further fixation with 1% osmium tetroxide in deionized water. Subsequent steps were performed as previously described. Electron micrographs were collected using TEM (HITACHI 7800, Tokyo, Japan) at 80 kV. The morphology of myocardial fibers and mitochondria was observed at ×14k, ×35k, and ×84k magnifications.

### Tissue ROS levels and ATP concentration measurement

ROS levels were measured by dihydroethidium (DHE) staining (Beyotime, Shanghai, China) of heart cryosections. The fluorescent images were analyzed with NIH ImageJ software. At least five fields per section were randomly chosen and analyzed. The ATP concentration in the myocardium was measured using an ATP assay kit (Beyotime, Shanghai, China) according to the protocol.

### Blood glucose measurement

The blood glucose level was measured using a glucometer (Omron, Kyoto, Japan). Blood was taken from the tail vein of each rat. The blood glucose levels of SD rats with different fasting times (0, 24, 48, 72 h and after descending) were recorded.

### Cell apoptosis and mitochondrial membrane potential (MMP) measurement

Apoptosis was analyzed using an Annexin V-FITC/PI apoptosis detection kit (BD Biosciences, NJ, USA) according to the manufacturer’s protocols. Apoptosis was evaluated using flow cytometry. The sum of early and late apoptotic cells was statistically analyzed.

MMP was measured using mitochondrial membrane potential assay kits (Beyotime, Shanghai, China). Cardiomyocytes were incubated with JC-1 solution and then evaluated using flow cytometry. The ΔΨm was quantified by the red/green fluorescence ratio.

### Immunofluorescence analysis of mitophagy in cardiomyocytes

To analyze autophagosomes, cells were transfected with GFP-LC3-adenovirus (Hanbio, Shanghai, China) as previously described. The cells were exposed to 1% O_2_ for 24 h after 24 h of transfection. After hypoxia treatment, cardiomyocytes were washed thoroughly with PBS, and immunofluorescent staining of BNIP3 was conducted. The cells were observed with a high‐content imaging system (Harmony, PerkinElmer, Germany). Images were collected from at least ten fields per well. The number of LC3 puncta and colocalization with BNIP3 protein were analyzed by NIH ImageJ software.

Double-staining of Tom20 and LAMP1 was performed to examine autolysosomes in mitochondria. After hypoxia treatment, cardiomyocytes were sequentially fixed with 4% paraformaldehyde, permeabilized with 0.1% Triton X-100, and blocked with goat serum. Then, the cells were coincubated with primary antibodies against Tom20 and LAMP1 overnight at 4 °C. Alexa Fluor 488-conjugated anti-mouse secondary antibodies, Alexa Fluor 594-conjugated anti-rabbit secondary antibodies, and DAPI were subsequently used. Images were collected from at least ten fields per well. The colocalization of Tom20 and LAMP1 was analyzed by ImagePro Plus software.

### Measurement of ROS and ATP in living cells

To determine the ROS level in living cells, adenoviruses carrying Cyto-RoGFP (Addgene plasmid #49435) or IMS-RoGFP (Addgene plasmid #49436) were used to examine ROS generation in the cytoplasm and mitochondrial intermembrane space (IMS), respectively. The principle of RoGFP has been previously described [6, 7]. Briefly, RoGFP was engineered with cysteine residues, enabling dithiol formation in response to oxidative stress. Oxidation resulting in dithiol formation causes RoGFP to be excited at 405 nm, while reduction causes RoGFP to be excited at 488 nm. Therefore, the ratio of 525 nm excitation at 405 nm to 525 nm excitation at 488 nm (fluorescence intensity of RoGFP) indicates the ROS levels in cells. Living cells in the Con and F groups were observed with a high‐content imaging system (Harmony, PerkinElmer, Germany) in 5% CO_2_ at 37 °C. The cells were then subjected to 1% O_2_ after the images were collected. After 1% O_2_ treatment for 24 h, the cells were immediately fixed, and the images were taken from the same fields of view. At least 20 cells per well were imaged.

The FRET-based fluorescent ATP probe MitGO-ATeam2 (provided by Hiromi Imamura, Kyoto University) was used to monitor mitochondrial ATP levels in living cells as previously described [6, 8]. MitGO-ATeam2 consists of an orange fluorescent protein (OFP), green fluorescent protein (GFP), and a sandwiched ε subunit of F0F1-ATPase. The conformation of the ε subunit can be changed by ATP binding, and therefore, the Förster resonance energy transfer (FRET) from 510 nm to 560 nm increases in MitGO-ATeam2-transfected cells. After a single excitation of 470 nm, the emission ratio of the 560 nm to 510 nm fluorescence intensity reflects the ATP level in cells. The MitGO-ATeam2 plasmid was transfected using Lipofectamine 3000 (Invitrogen, CA, USA). The procedures were conducted in accordance with the manufacturer’s instructions. The images were taken using the same manner as detailed above.

### Measurement of the oxygen consumption rate (OCR) and extracellular acidification rate (ECAR) of cardiomyocytes

An XF24 extracellular flux analyzer (Agilent Seahorse Bioscience, CA, USA) was used to measure the OCR and ECAR as previously described [9]. Cardiomyocytes were seeded in *Seahorse* XF24 microplates at a density of 10^4^ cells/well. After treatments, the cells were cultured with XF assay medium in a 37 °C incubator without CO_2_ for 1 h before being assayed. Basal respiration was measured before the addition of oligomycin. Proton leak-linked respiration was recorded after oligomycin was added. The increase in the oxygen consumption of the cells after treatment with FCCP indicates the maximal respiratory capacity of these cells. Finally, antimycin A and rotenone were used to evaluate the spare respiratory capacity. Measurement of ECAR was performed in glucose-deprived media with the successive addition of glucose, oligomycin, and 2-deoxyglucose. The OCR and ECAR values were normalized to the protein concentration of the cells and are presented as pmol/min/μg protein and mpH/min/μg protein, respectively.

### siRNA transfection

For transfection, 2 × 10^5^ cardiomyocytes were seeded per well in a six-well plate, 2 μg of siRNA (HanBio, Shanghai, China) and 10 μL of X-tremeGENE siRNA transfection reagent (Roche, Basel, Switzerland) were diluted separately in Opti-MEM, and then the siRNA-X-treme reagent complex solution was added to the cells. The cells were incubated for 36 h, after which further treatment was performed. The siRNA sequences were as follows: mTOR-siRNA sense: 5′-GCUAGAAGCCUUUGUCUAUTT-3′ and mTOR-siRNA antisense: 5′- AUAGACAAAGGCUUCUAGCTT-3′; and NC-siRNA (negative control) sense: 5′-UUCUCCGAACGUGUCACGUTT-3′ and NC-siRNA antisense: 5′-ACGUGACACGUUCGGAGAATT-3′.

### Statistical analysis

All experiments were independently repeated at least three times, and the data are presented as the mean ± SD. Significance was determined using one-way ANOVA followed by Tukey’s multiple comparison test with GraphPad Prism 8.0 (GraphPad Software, La Jolla, California, USA). A value of *P* < 0.05 was considered significant.

## Results

### Fasting improved the survival rates of rats and alleviated tissue injuries during acute extreme hypoxia

Supplementary Table S3 shows the survival rates of rats with different fasting pretreatment times after exposure to 7620 m for 24 h. The rat survival rate gradually increased with the extension of the fasting pretreatment time and was 88.5% for 72 h of fasting. Clearly, fasting pretreatment for 72 h improved the rat survival rate most prominently and was chosen as the optimal time.

**Fig. 1:**
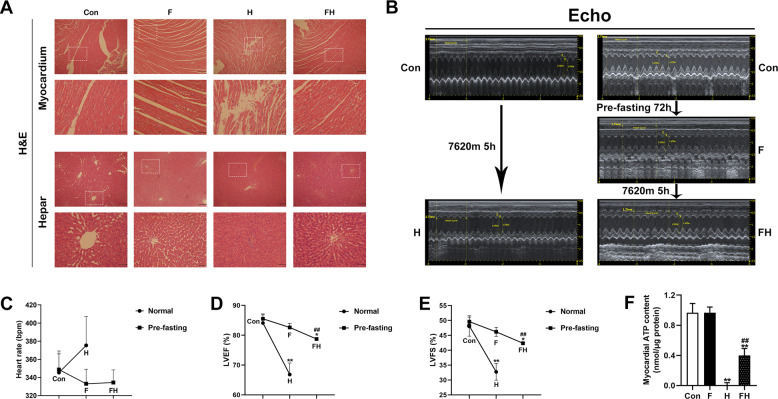
Fasting pretreatment alleviated tissue injuries and maintained cardiac function during acute extreme hypoxia. **A** Histological staining (hematoxylin and eosin) of myocardial and hepatic tissue, scale bar = 200 μm and 50 μm (enlarged diagram). The results are representative of 5 rats/group. **B** Representative images of myocardial M-mode echocardiography images. SD rats were subjected to echocardiography in the following stages: before hypoxia treatment and after exposure to 7620 m for 5 h (Con-CH groups); before 72 h of fasting, after 72 h of fasting, namely, before hypoxia treatment and after exposure to 7620 m for 5 h (Con-F-FH groups). **C**−**E** Quantitative analysis of heart rate (HR), left ventricular ejection fraction (LVEF), and left ventricular fractional shortening (LVFS). The values are presented as the mean ± SEM (*n* = 3 rats/group). **F** Statistical analysis of myocardial tissue ATP levels (nmol/μg protein) (*n* = 5 animals/group). The values are presented as the mean ± SEM. *P < 0.05 or **P < 0.01 vs. the Con group. ^##^*P* < 0.01 vs. the H group. See also Supplementary Table S3 and Supplementary Fig. S1.

The H&E staining results are shown in Fig. 1A. Cardiac tissue was badly damaged in the H group relative to the Con group, as indicated by ruptured cardiomyocytes and disordered myocardial fibers. The hepatic tissue of the H group was also impaired, exhibiting a disruption in hepatic lobule structure and irregular liver cell morphology. However, these impaired features were alleviated with fasting pretreatment in the FH group. Cardiac function was assessed by echocardiography (Fig. 1B). After 7620 m for 5 h, the LVEF and LVFS were significantly decreased. Fasting pretreatment prevented cardiac function impairment caused by acute extreme hypoxia, as evidenced by the abrogated reductions in LVEF and LVFS in the FH group (Fig. 1C−E).

Additionally, ROS generation was significantly increased in H-group hearts compared with Con-group hearts and was obviously higher than that in FH-group hearts (Supplementary Fig. S1A, S1B). Direct ATP analysis revealed that myocardial ATP was almost exhausted in the myocardium in the H group, while this outcome was modified in the FH group (Fig. 1F).

### Fasting reduced tissue protein synthesis and lipogenesis during acute extreme hypoxia

**Fig. 2:**
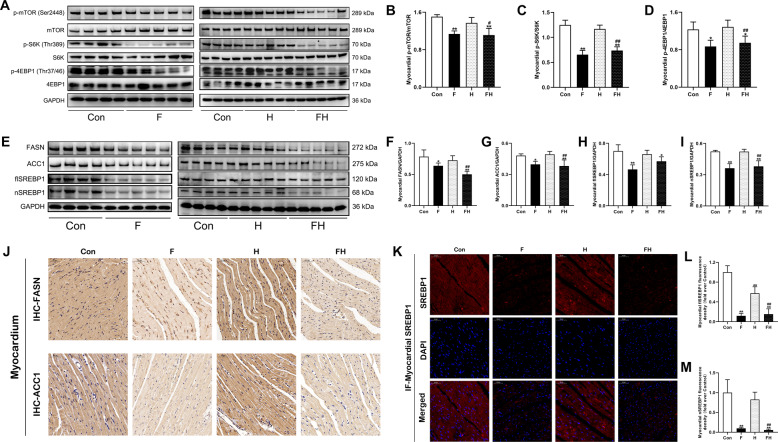
Fasting pretreatment reduced tissue anabolism during acute extreme hypoxia. **A**−**D** Representative western blot bands and quantitative analysis of protein synthesis-related proteins in the myocardium. **E**−**I** Representative western blot bands and quantitative analysis of lipogenesis-related proteins in the myocardium. **J** Representative immunohistochemical staining of FASN and ACC1 in the myocardium. Scale bars = 50 μm. **K** Representative immunofluorescent images of the myocardium stained for SREBP1 (red) and Hoechst 33258 (blue). The red fluorescence represents full-length, cytoplasm-localized SREBP1 (flSREBP1), and the violet fluorescence shows active, nuclear-localized SREBP1 (nSREBP1). Scale bars = 50 μm. **L**, **M** Quantitative analysis of flSREBP1 (red) and nSREBP1 (violet) fluorescence intensities in heart sections. In (**A**) and (**E**), the samples were derived from the same experiment, and gels/blots were processed in parallel. GAPDH was used as a loading control. The values are presented as the mean ± SEM (*n* = 5 animals/group). *P < 0.05 or **P < 0.01 vs. the Con group. ^#^*P* < 0.05 or ^##^*P* < 0.01 vs. the H group. See also Supplementary Fig. S2.

In cardiac and liver tissues, as shown in Fig. 2A−D and Supplementary Fig. S2A−S2D, the levels of phospho-Ser2448-mTOR, phospho-Thr389-S6K, and phospho-Thr37/46–4EBP1 were decreased in the F and FH groups compared to the Con group, and the decreases were more pronounced in the FH group than in the H group. These results indicated that mTOR activity and protein synthesis were reduced after fasting pretreatment for 72 h.

Next, lipogenic processes were investigated in cardiac and liver tissues. As shown in Fig. 2E−I and Supplementary Fig. S2E−S2I, FASN and ACC1 expression were substantially lower in the F and FH groups than in the Con group. Both flSREBP1 and nSREBP1 protein levels were reduced in the F and FH groups. In addition, the expression of these lipogenesis-related proteins in the FH group was markedly decreased compared to that in the H group. The expression of FASN and ACC1 was analyzed via immunohistochemistry and exhibited a similar trend as was observed by western blotting (Fig. 2J and Supplementary Fig. S2J). The immunofluorescence results suggested that the flSREBP1 and nSREBP1 fluorescence intensities in the F and FH groups were significantly reduced compared with those in the Con group, and the nSREBP1 fluorescence intensity was markedly lower than that in the H group (Fig. 2K–M and Supplementary Fig. S2K−S2M). Furthermore, although the lipid droplets in liver tissue in the F, H, and FH groups were fewer than those in the Con group, the levels in the FH group were significantly lower than those in the H group (Supplementary Fig. S2N−S2O).

### Fasting enhanced BNIP3-mediated mitophagy in the myocardium during acute extreme hypoxia

**Fig. 3:**
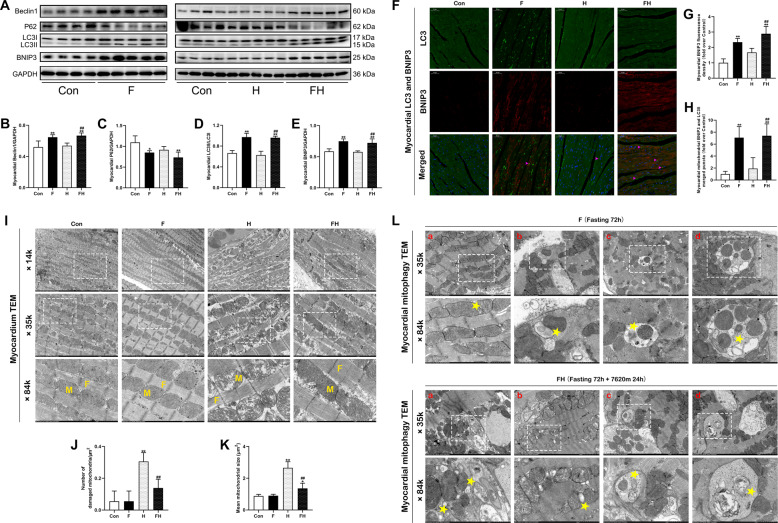
Fasting pretreatment enhanced myocardial mitophagy levels in the myocardium under acute extreme hypoxia. **A**−**E** Representative western blots and quantitative analysis of autophagy-related proteins (Beclin1, P62, LC3) and a mitophagy-related protein (BNIP3) in the myocardium. The samples were derived from the same experiment, and gels/blots were processed in parallel. **F** Representative immunofluorescent images of the myocardium after double-staining for LC3 (green) and BNIP3 (red). The yellow puncta formed by the merging of green and red fluorescence indicated BNIP3-mediated mitophagy, as indicated by pink arrows and shown in the merged row. Scale bars = 50 μm. **G**, **H** Quantitative analysis of BNIP3 (red) fluorescence intensity and the number of merged LC3-BNIP3 puncta (yellow). **I** Representative electron micrograph showing myocardial fibers and mitochondrial shape. The white-boxed regions are enlarged in the next row. M represents mitochondria, and F represents myocardial fibers in the ×84K row. Scale bars = 5 μm (×14k), 2 μm (×35k), 1 μm (×84k). **J**, **K** Quantitative analysis of the number of damaged mitochondria per μm^2^ and the mean size of mitochondria. **L** Representative electron micrograph showing different stages of mitophagy in the myocardium in the F and FH groups, as indicated by yellow pentagrams. The white-boxed regions are enlarged in the 84K row. Scale bars = 2 μm (×35k), 1 μm (×84k). **a** Membrane vesicles in close apposition to the OMM. **b** Membrane vesicles wrapping around mitochondria. **c** An intact mitochondrion can be seen inside an autophagosome. **d** Autolysosomes containing digested material and mitochondria. The values are presented as the mean ± SEM (*n* = 5 animals/group). *P < 0.05 or **P < 0.01 vs. the Con group. ^#^*P* < 0.05 or ^##^*P* < 0.01 vs. the H group.

As shown in Fig. 3A–E, the expression of Beclin1 and LC3II/I was significantly increased and the expression of P62 was decreased in the myocardium of the F and FH groups; the elevated expression of Beclin1 and LC3II/I in the FH group was much higher than that in the H group. The level of BNIP3 was upregulated in the F and FH groups and higher than that in the H group. Immunofluorescence staining was used to detect the colocalization of BNIP3 and LC3. The yellow puncta showing the colocalization of LC3 puncta and BNIP3 represent BNIP3-mediated mitophagy. Clear LC3 puncta appeared in the F and FH groups but were hardly observed in the Con and H groups (Fig. 3F and Supplementary Fig. 3H). Quantitative analysis showed that the fluorescence intensity of BNIP3 was significantly increased in the F and FH groups and was higher than those in the H group (Fig. 3G). Representative electron micrographs of the myocardium are shown in Fig. 3I. The myocardium of the H group showed severe disorganization, fractured myocardial fibers, and massive swollen mitochondria with disrupted cristae and ruptured mitochondrial membranes, which was verified by the increased number of damaged mitochondria and increased size of mitochondria (Fig. 3J, K). The myocardium of the FH group showed slight injury compared to that of the H group. Many autophagic vacuoles and other autophagic structures around the mitochondria were observed in the F and FH groups as shown in Fig. 3L.

### Simulated fasting protected against acute hypoxic injuries in cardiomyocytes

The glucose level gradually declined with the extension of the fasting duration and eventually stabilized at approximately half of the initial level (Supplementary Fig. S3A). The vitro results revealed that as glucose concentrations in the culture medium increased, intracellular p-mTOR levels increased (Supplementary Fig. S3B−3D). Consequently, cardiomyocytes were routinely cultured in normoglycemic (1 g/L glucose) DMEM and switched to hypoglycemic (0.5 g/L glucose + 0.5 g/L mannitol) DMEM for 72 h to simulate the effect of fasting pretreatment on mTOR in vitro.

Compared with that in the Con group, the cleaved/pro-caspase3 level was obviously increased in the H group, while there was no significant difference in the FH group (Supplementary Fig. S4A−S4B). Additionally, the flow cytometric results showed that low glucose pretreatment for 72 h notably reduced apoptosis and alleviated the decrease of MMP in the FH group compared to the H group (Supplementary Fig. S4C−S4F).

### Simulated fasting reduced protein synthesis and lipogenesis in cardiomyocytes under acute hypoxia

**Fig. 4:**
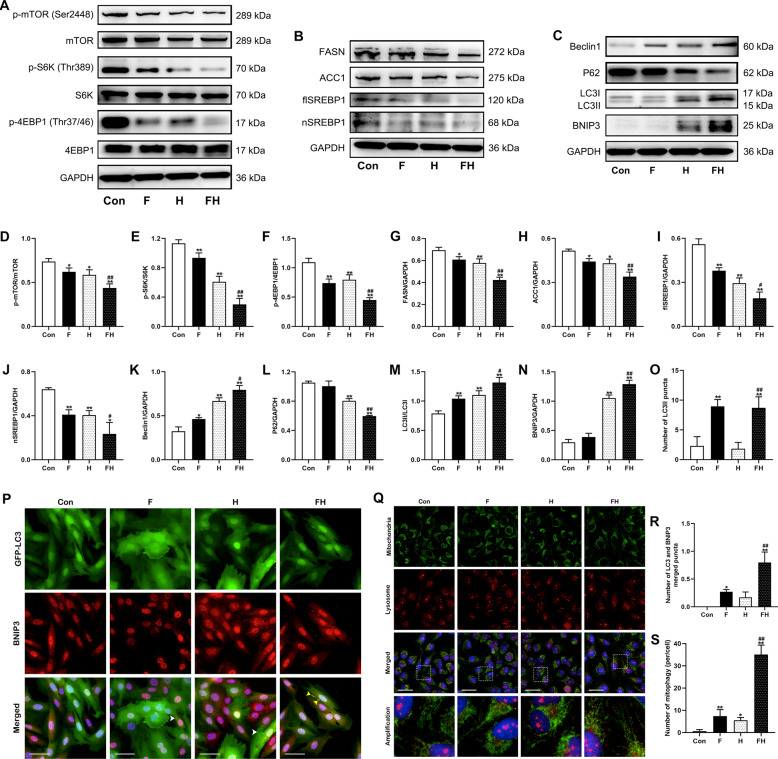
Simulated fasting weakened cellular anabolism and enhanced BNIP3-mediated mitophagic flux in cardiomyocytes under acute hypoxia. **A**, **D**−**F** Representative immunoblots (**A**) and quantification (**D**−**F**) of protein synthesis-related protein (p-mTOR/mTOR, p-S6K/S6K, and p-4EBP1/4EBP1) levels in cardiomyocytes. **B**, **G**−**J** Representative immunoblots (**B**) and quantification (**G**−**J**) of lipogenesis-related protein (FASN, ACC1, flSREBP1, and nSREBP1) levels in cardiomyocytes. **C**, **K**−**N** Representative immunoblots (**C**) and quantification (**K**−**N**) of autophagy-related proteins (Beclin1, P62, LC3) and a mitophagy-related protein (BNIP3) in cardiomyocytes. **O**, **P**, **R** Representative immunofluorescent images (**P**) of the colocalization of GFP-LC3 puncta (green) and BNIP3 protein (red) in cardiomyocytes with stable GFP-LC3 expression. Scale bars = 50 μm. Quantitative analysis (**O**, **R**) of GFP-LC3 puncta (white arrow) and merged GFP-LC3/BNIP3 puncta (yellow arrow). **Q**, **S** Representative immunofluorescent images and quantification of the colocalization of mitochondria (green, marked by Tom20) and lysosomes (red, marked by LAMP1) in cardiomyocytes. Amplification and higher-magnification views of the boxed regions in the merged column. Scale bars = 20 μm. In (**A**−**C**), the data represent the mean ± SEM of three independent experiments. In (**P**) and (**Q**), the results are representative of five independent experiments, five fields each. *P < 0.05 or **P < 0.01 vs. the Con group. ^#^*P* < 0.05 or ^##^*P* < 0.01 vs. the H group.

The western blotting results showed that the levels of p-mTOR, p-S6K, and p-4EBP1 were all decreased in the F, H, and FH groups. Among the three treatment groups, the reductions in p-mTOR, p-S6K, and p-4EBP1 expression were most prominent in the FH group and were lower than those in the H group (Fig. 4A–F). Then, lipogenesis-related proteins were analyzed (Fig. 4B). The expression of FASN, ACC1, SREBP1 was decreased in the F, H, and FH groups. Moreover, the FH group showed the lowest protein levels among the three groups (Fig. 4G–J).

### Simulated fasting promoted BNIP3-mediated mitophagy in cardiomyocytes under acute hypoxia

The expression levels of Beclin1 and LC3II/I were increased in the F, H, and FH groups. The increases in the FH group were much higher than those in the H group. P62 expression was decreased and BNIP3 expression was obviously increased in the H and FH groups compared to the Con group. The FH group showed the most pronounced changes in P62 and BNIP3 expression, with a significant difference compared with that in the H group (Fig. 4C and Supplementary Fig. 4K–N).

As shown in Fig. 4P, the colocalization of GFP-LC3 puncta and BNIP3 indicates the formation of autophagosomes. The number of LC3 puncta and merged LC3-BNIP3 puncta were quantified. The numbers of LC3 puncta and merged LC3-BNIP3 puncta in the F and FH groups were both increased compared with those of the Con group, and the FH group had the highest number of merged LC3-BNIP3 puncta (Fig. 4O–4R). Furthermore, the autolysosomes were detected (Fig. 4Q). Quantitative analysis showed that the number of autolysosomes per cell was increased in the F, H, and FH groups. Cells in the FH group had a higher number of autolysosomes than that of the H group (Fig. 4S).

### Simulated fasting improved mitochondrial oxygen utilization efficiency and promoting ATP production in cardiomyocytes under acute hypoxia

**Fig. 5:**
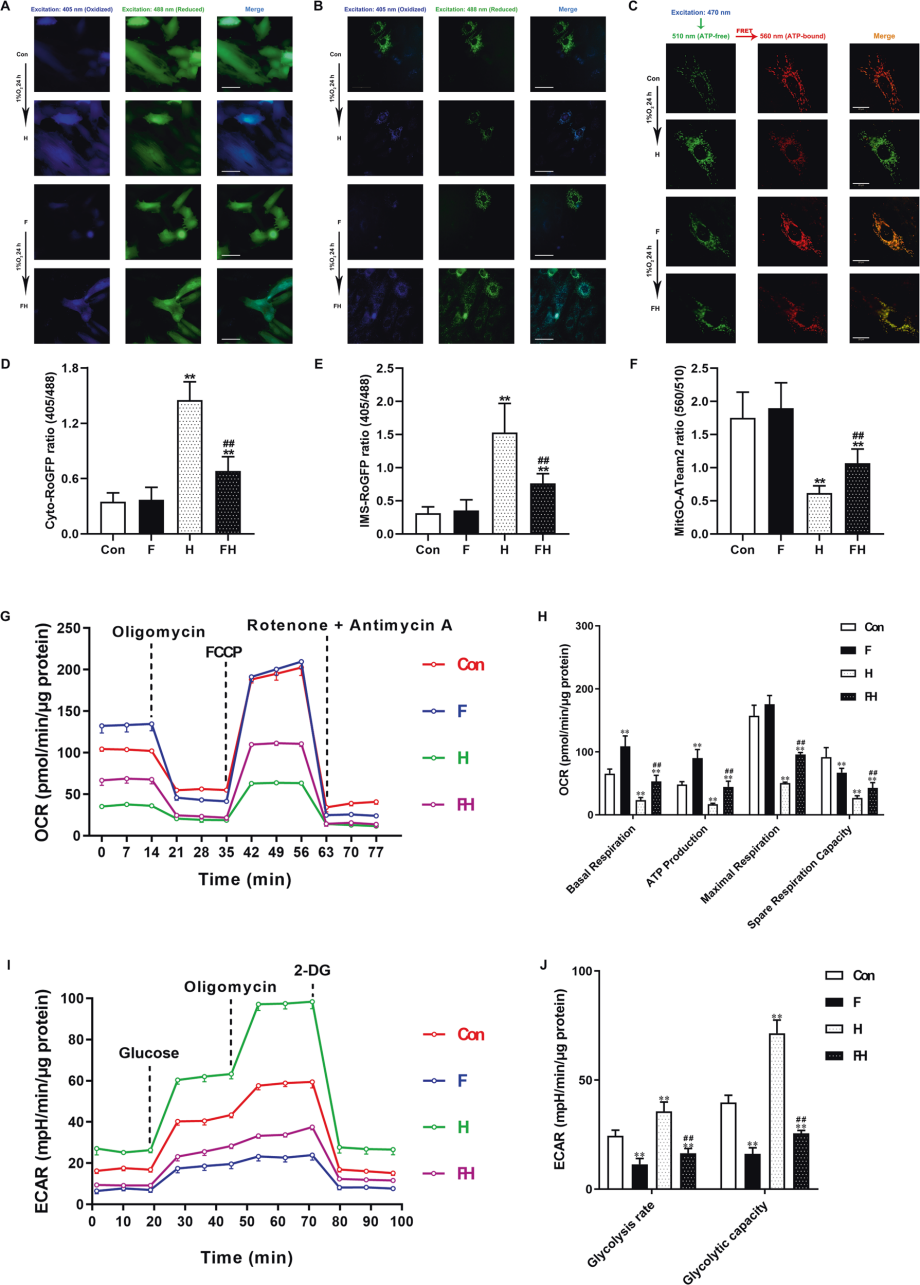
Simulated fasting reduced ROS production, restored ATP production, and increased OCR levels in cardiomyocytes during acute hypoxia. **A**, **B** Representative immunofluorescent images of cardiomyocytes at different stages after transfection with Cyto-RoGFP or IMS-RoGFP. Excitation at 405 or 488 nm indicates oxidation or reduced states, respectively. The ratio (405−488 nm) reflects the redox status of the indicated site. Scale bars = 50 μm. **C** Representative immunofluorescent images of cardiomyocytes transfected with MitGO-ATeam2 at different stages. The mitochondrial ATP level was indicated by the ratio (emission at 560−510 nm). Scale bars = 50 μm. **D**, **E** Quantitative analysis of the Cyto-RoGFP and IMS-RoGFP (405−488 nm) ratios. **F** Histogram of the MitGO-ATeam2 ratio (560−510 nm). **G**, **H** Line graph and statistical analysis of OCR parameters, including basal respiration, ATP production, maximal respiration, and spare respiration. **I**, **J** Line graph and statistical analysis of ECAR parameters, including glycolysis rate and glycolytic capacity. In (**A**−**C**), the data are the means ± SEM, *n* > 20 fields from three independent experiments. **P < 0.01 vs. the Con group. ^##^*P* < 0.01 vs. the H group.

As shown in Fig. 5A, B, the ROS levels of cytosol and IMS were evaluated. The results showed that simulated fasting had no effect on ROS generation in the cytosol or IMS. Despite the slightly elevated ROS levels in cytosol and IMS compared to those in the Con group, the FH groups had notably lower ROS levels than that in the H group (Fig. 5D, E). The results of MitGO-ATeam2 showed that simulated fasting had no significant effect on mitochondrial ATP production. ATP production was significantly decreased in the H group, while this reduction was ameliorated in the FH group (Fig. 5C, F). The OCR and ECAR of cardiomyocytes were measured (Fig. 5G, I). The basal and ATP-linked respiration were significantly improved in the F group. Acute hypoxia seriously affected mitochondrial respiratory capacity, which was characterized by suppressed basal respiration, ATP-linked respiration, maximal respiration, and spare respiration, all of which were significantly improved in the FH group (Fig. 5H). Besides, the cardiomyocytes in the H group showed a drastic increase in both glycolysis and glycolytic capacity compared to control cells. Fasting pretreatment obviously inhibited the glycolysis process of cells in the F and FH groups (Fig. 5J).

### mTOR knockdown prevented acute hypoxic injuries in cardiomyocytes

As shown in Supplementary Fig. S5A and S5B, cleaved/pro-caspase3 was increased in the si-NC + H group, while there was no significant difference in the si-mTOR + H group compared to the si-NC group. The apoptotic rate of cells in the si-mTOR + H group showed no difference from that of the si-NC group and was markedly lower than that in the si-NC + H group (Supplementary Fig. S5C−S5D). Additionally, MMP analysis showed that mTOR knockdown preserved MMP after hypoxia treatment in the si-mTOR + H group (Supplementary Fig. S5E−S5F).

### mTOR knockdown attenuated anabolism and increased BNIP3-mediated mitophagy in cardiomyocytes under acute hypoxia

**Fig. 6:**
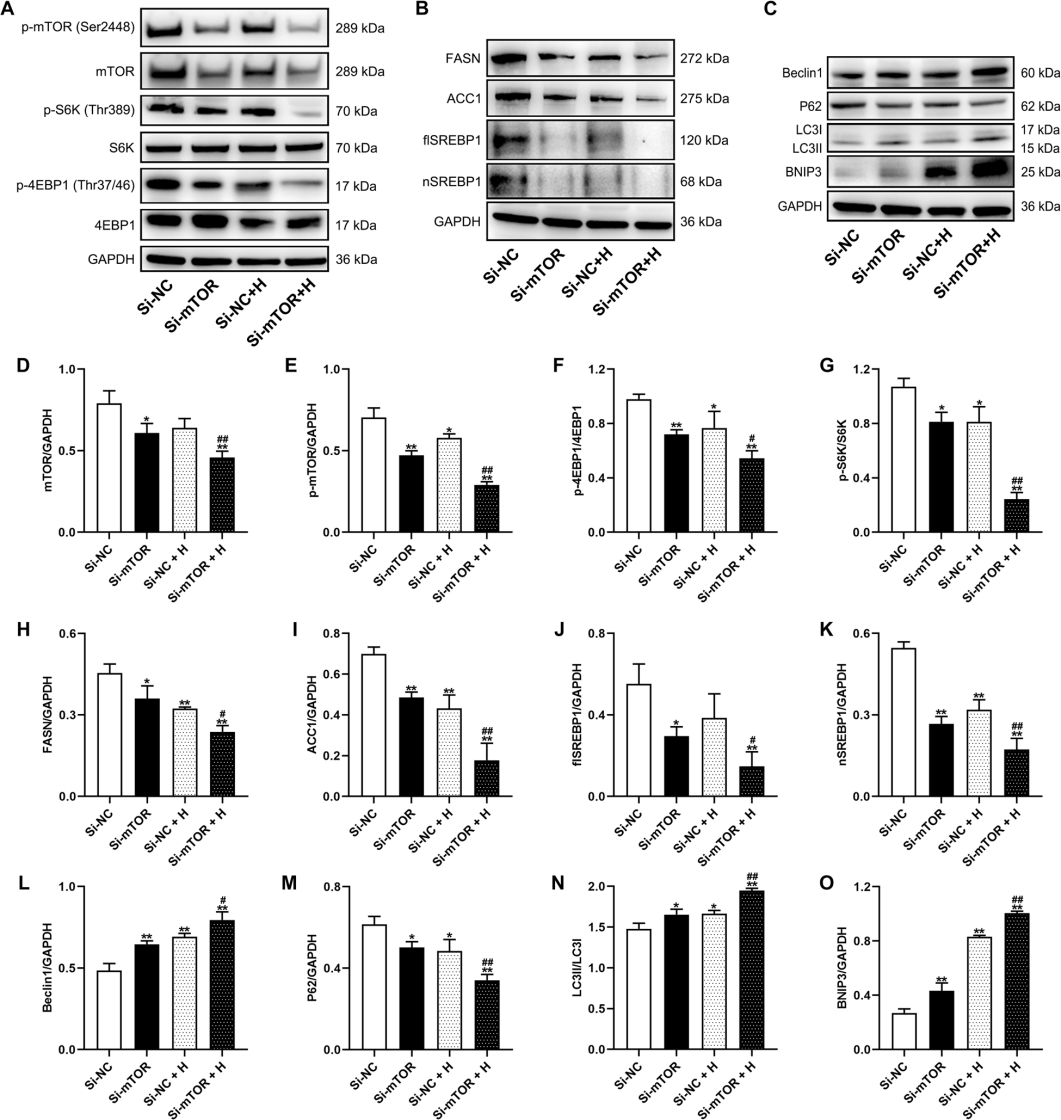
mTOR gene interference attenuated cellular anabolism and improved BNIP3-mediated mitophagic flux in cardiomyocytes under acute hypoxia. **A**, **D**−**G** Representative immunoblots and quantification of protein synthesis-related protein (p-mTOR/mTOR, p-S6K/S6K, and p-4EBP1/4EBP1) levels in cardiomyocytes. **B**, **H**−**K** Representative immunoblots and quantification of lipogenesis-related protein (FASN, ACC1, flSREBP1, and nSREBP1) levels in cardiomyocytes. **C**, **L**−**O** Representative immunoblots and quantification of autophagy-related proteins (Beclin1, P62, LC3) and a mitophagy-related protein (BNIP3) in cardiomyocytes. The values are presented as the mean ± SEM (*n* = 3). *P < 0.05 or **P < 0.01 vs. the Si-NC group. ^#^*P* < 0.05 or ^##^*P* < 0.01 vs. the Si-NC + H group.

Compared to the si-NC group, cardiomyocytes treated with si-mTOR showed apparent reductions in p-mTOR and mTOR, as well as significantly reduced the levels of p-S6K and p-4EBP1. The si-mTOR + H group showed the most prominent decreases in p-mTOR, p-S6K, and p-4EBP1 expression, which were also obviously lower than those of the si-NC + H group (Fig. 6A, D–G). Furthermore, lipogenesis-related proteins expression was significantly decreased in the si-mTOR group and further reduced in the si-mTOR+H group, which was significantly lower than that in the si-NC + H group (Fig. 6B, H–K).

The expression of beclin1 and LC3II/I was upregulated and P62 was downregulated in the si-mTOR group. The changes were more notable in the si-mTOR + H group. Notably, the expression of BNIP3 was significantly increased in the si-mTOR group. Hypoxia obviously upregulated BNIP3 expression in both the si-NC + H and si-mTOR + H groups. The BNIP3 level in the si-mTOR + H group was highest and higher than that in the si-NC + H group (Fig. 6C, L–O).

### mTOR knockdown improved mitochondrial oxygen utilization efficiency and promoting ATP production in cardiomyocytes under acute hypoxia

**Fig. 7:**
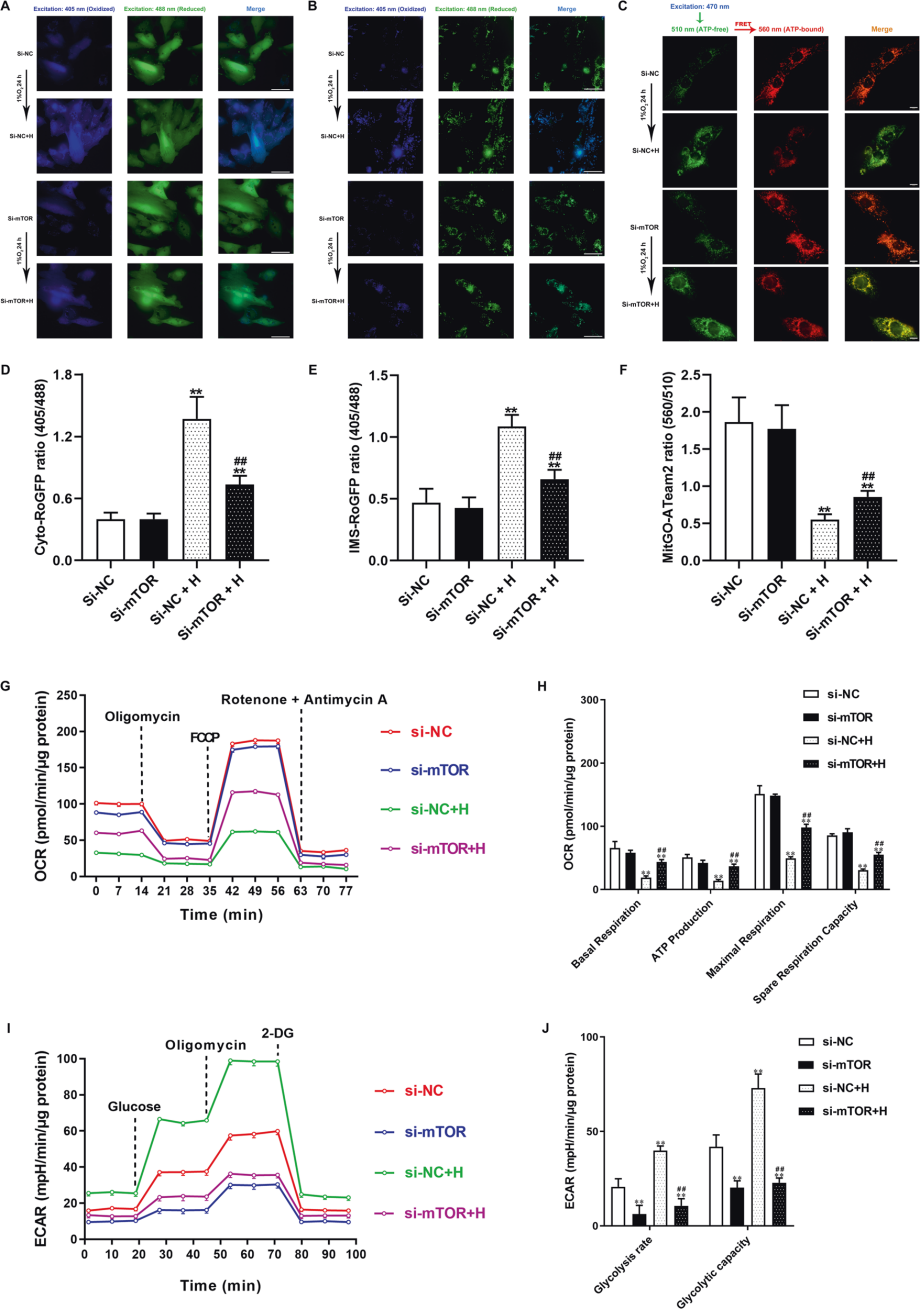
mTOR gene interference inhibited ROS production, preserved ATP generation, and increased OCR levels under acute hypoxia. **A**−**C** Representative immunofluorescent images of cardiomyocytes transfected with RoGFP or MitGO-ATeam2 at different stages. Scale bars = 50 μm. **D**−**F** Quantitative analysis of the RoGFP (405- to 488-nm) ratio and MitGO-ATeam2 ratio (560- to 510-nm). **G**, **H** OCR variation curves and statistical diagram showing OCR parameters. **I**, **J** ECAR variation curves and statistical diagram showing ECAR parameters. In (**A**−**C**), the data are the mean ± SEM, *n* > 20 fields of three independent experiments. **P < 0.01 vs. the Si-NC group. ^##^*P* < 0.01 vs. the Si-NC + H group.

Similarly, the ROS and ATP in cardiomyocytes were monitored (Fig. 7A–C). The results showed that si-mTOR treatment had no prominent effect on cytoplastic and IMS ROS; however, cytoplastic and IMS ROS levels were significantly decreased in the si-mTOR + H group compared to the markedly increased ROS levels in the si-NC + H group (Fig. 7D, E). Moreover, the ATP level in cardiomyocytes in the si-NC + H group was markedly decreased compared to that in the si-NC group and was obviously ameliorated in the si-mTOR + H group (Fig. 7F). OCR parameters, including the decreased basal respiration, ATP production, maximal respiration, and spare respiration, were significantly restored after mTOR knockdown under acute hypoxia (Fig. 7G–H). ECAR parameters, including glycolysis rate and glycolytic capacity, were drastically decreased in mTOR-knockdown cells of si-mTOR and si-mTOR + H groups (Fig. 7I–J).

## Discussion

**Fig. 8:**
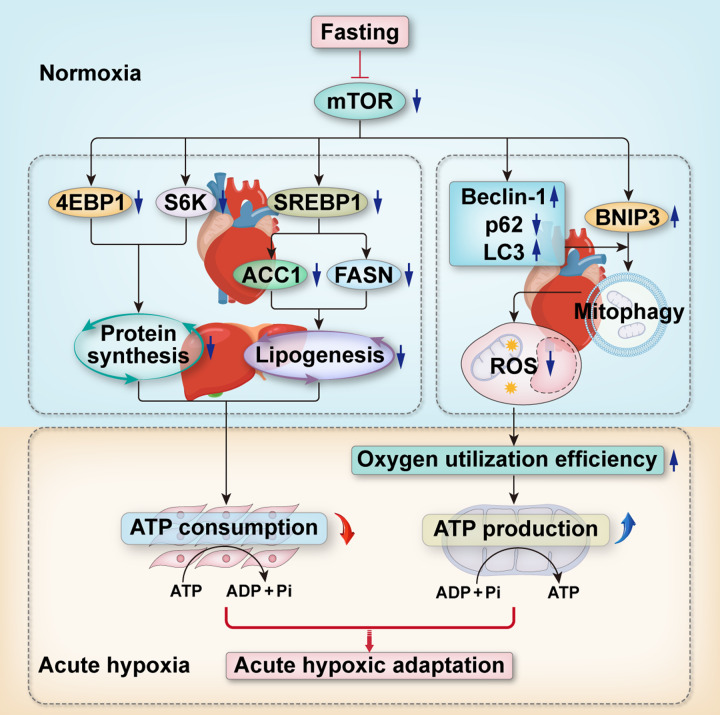
Schematic diagram illustrating that fasting pretreatment enhances the adaptability to acute hypoxia by alleviating anabolism and promoting mitophagy via inhibiting mTOR. On the one hand, the inhibited mTOR induced by fasting reduces the level of protein synthesis by decreasing the expression of 4EBP1 and S6K and reduces lipogenesis by decreasing the transcription of ACC1 and FASN genes via inhibiting the expression of SREBP1. On the other hand, by regulating autophagy-related proteins Beclin1, p62, and LC3 and increasing the expression of mitophagy receptor protein BNIP3, the inhibited mTOR enhances mitophagy, contributing to reduce the generation of mitochondrial-derived ROS, thus improving the oxygen utilization efficiency of mitochondria under acute hypoxia. In short, through fasting pretreatment, the ATP consumption is decreased and the ATP production is increased under acute hypoxia, so that the body can quickly adapt to acute hypoxic environment.

This study is the first to demonstrate that fasting pretreatment for 72 h can be used as an effective strategy for rapid adaptation to acute extreme hypoxia. Mechanistically, fasting inhibited mTOR activity by inducing hypoglycemia, which acted in two ways. On the one hand, mTOR inhibition attenuated the levels of protein synthesis and lipogenesis, which, in turn, prevented the unnecessary consumption of cellular ATP; on the other hand, mTOR inhibition activated BNIP3-mediated mitophagy to eliminate damaged mitochondria, which then reduced the production of mitochondrial ROS and improved mitochondrial oxygen utilization efficiency to ultimately increase ATP production under hypoxic conditions (Fig. 8). Thus, the acute hypoxia endurance capacity of rats was increased. Overall, our study proved that mTOR, as a key molecule by which fasting exerts protective effects, plays a crucial role in improving the body’s adaptability to acute extreme hypoxia by regulating cellular anabolism and mitophagy.

Altitudes above 7620 m are considered death zones, in which no man can adapt or remain for a long period of time [10]. Studies have shown that alveolar oxygen partial pressure drops to the critical value of 30 mmHg at 7620 m [11, 12]. Therefore, the effective consciousness time of humans at 7620 m is generally accepted to determine acute hypoxia tolerance at high altitudes [11]. The survival rates of rats after remaining at 7620 m for 24 h were used as the judgment index [10].

### Fasting prevents hypoxic injuries by inhibiting mTOR activity

We first confirmed that fasting significantly inhibits the activity of mTOR in vivo. Moreover, fasting reduced tissue injuries and improved cardiac function under acute extreme hypoxia, verified by H&E staining and echocardiography. We further used 0.5 g/L glucose to inhibit the mTOR activity of cardiomyocytes and simulate the effect of fasting. The in vivo and in vitro experimental results were consistent. Simulated fasting pretreatment significantly reduced the acute hypoxic injuries of cardiomyocytes, including lessening the activity of caspase3, preserving the MMP, and decreasing apoptosis rate. Finally, we demonstrated that mTOR is the key factor through which fasting exerts a protective effect during acute hypoxia by knocking down the level of cellular mTOR. mTOR knockdown enhanced the survival of cardiomyocytes under acute hypoxia, as indicated by the decrease in caspase-3 activity, restoration of MMP, and reduction in the apoptotic rate of cells.

### Fasting attenuates anabolism to reduce unnecessary ATP consumption by inhibiting mTOR

mTOR regulates cell growth by maintaining the balance between anabolism and catabolism by sensing intracellular nutritional status. The mTORC1 complexes regulate protein synthesis by phosphorylating the key translation-related factors S6K and 4EBP1 [13]. SREBP1, a major transcriptional regulator of lipid metabolism-related genes, exists as an inactive precursor form and an active cleaved form within cells [14]. The precursor of SREBP1 (flSREBP1) is processed and matured in the Golgi and transferred to the nucleus (nSREBP1) to regulate the transcription of lipogenesis-related genes such as ACC1 and FASN [14, 15]. mTOR promotes lipogenesis by increasing the expression of SREBP1 and further accumulation in the nucleus [16]. Moreover, mTOR also participates in the regulation of autophagy [17]. At present, many studies have shown that in a variety of tumors, the mTOR level of tumor tissues and its upstream and downstream molecules are changed [18–20]. Consequently, mTOR has become an effective target for some tumor therapies [21, 22]. In addition to playing a role in a large number of tumors, whether mTOR is involved in adaptability to acute extreme hypoxia aroused our interest.

Combining the above results, we hypothesized that fasting preconditioning affected tissue energy reserves by regulating tissue metabolism by inhibiting mTOR activity, which may be the potential mechanism by which fasting pretreatment exerts protective effects under acute extreme hypoxia. Our results indicated that fasting-pretreated inhibited the levels of protein synthesis and lipogenesis in both rat tissues and H9C2 cells. Interestingly, the levels of protein synthesis-related and lipogenesis-related proteins in normal cardiomyocytes were significantly decreased after 24 h of acute hypoxia. This result also indirectly verifies our idea that normal cells exposed to acute hypoxia will compensatively reduce their levels of anabolism to reduce unnecessary ATP consumption. Fasting pretreatment exerts a protective effect by strengthening this compensatory response. In cardiomyocytes preconditioned with simulated fasting, protein synthesis and lipogenesis were inhibited most significantly after acute hypoxia. Furthermore, in mTOR-knockdown cells, protein synthesis and lipogenesis were also inhibited under acute hypoxia.

The synthesis of glycogen, fatty acids, cholesterol, and proteins consumes ATP, especially the synthesis of high-molecular-weight proteins. The amount of ATP consumed during the entire process of protein or fatty acid synthesis cannot be underestimated. Under an acute hypoxic environment, reducing ATP consumption by unnecessary cellular activities to ensure the activity of vital organs may be an important strategy to guarantee the survival of the body. Our study confirmed that fasting pretreatment weakened the activity of mTOR and then reduced the expression of proteins related to protein synthesis and lipogenesis, preventing unnecessary ATP consumption under acute hypoxia.

### Fasting enhances mitochondrial oxygen utilization efficiency through mitophagy by inhibiting mTOR under acute hypoxia

The electron transport chain (ETC) of mitochondria, in which electrons are passed from substrates to O_2_ for ATP production, provides nearly 90% of the ATP necessary for cellular activity [23]. During the transfer of electrons from metabolic substrates to O_2_ via the ETC, some electrons will overflow the ETC and be directly captured by O_2_ to produce ROS. Currently, the finding that acute hypoxia induces the generation of redundant intracellular ROS has been universally recognized [24]. There is no doubt that the formation of ROS will lead to the consumption of oxygen, which in turn leads to less O_2_ being used for ATP production. Therefore, the oxygen utilization efficiency of mitochondria declines under acute hypoxia. Our previous study showed that acute hypoxia depresses ATP production and increases oxygen consumption as a result of enhanced ROS generation in mitochondria [6]. That means the mitochondrial oxygen utilization efficiency (utilizing O_2_ to produce ATP) can be improved to some extent by reducing the generation of ROS.

A variety of key mechanisms are involved in mitochondrial quality control, including mitochondrial fission and fusion, mitophagy, and mitochondrial biogenesis [25, 26]. Mitophagy, the selective autophagy of mitochondria, is induced to degrade senescent and dysfunctional mitochondria. BNIP3 is a transmembrane protein in the outer mitochondrial membrane that contains an LIR motif region on the cytoplasmic side, which can be recognized by LC3 to form autophagosomes, engulfing mitochondria [27]. Previous evidence showed that the protein expression of BNIP3 is upregulated under hypoxic conditions, which enhances mitophagy and protects cells against ROS accumulation [28]. However, there is no change in p-mTOR and autophagy-related proteins in the myocardium of the H group in our vivo experiments. We considered that the cells of myocardium had been forced to death due to severe hypoxic injury before the p-mTOR inhibition and autophagy activation since the rats could only survive 4−6 h at 7620 m altitude. Our results confirmed that fasting pretreatment significantly increased the BNIP3-mediated mitophagy. In addition, we found that knocking down mTOR directly increased the expression level of BNIP3 in cells. Previous studies showed that BNIP3 interacts with Rheb and then inhibits mTOR activity under hypoxia [29]. How mTOR inhibition increases the expression of BNIP3 needs to be further explored.

The results of ROS analysis in tissue and cell showed that fasting can reduce the increase in ROS during acute hypoxia, suggesting that enhanced mitophagy protects cells from oxidative stress, which was consistent with a previous study. In addition, knocking down mTOR reduced the generation of cellular ROS. The ATP level in the fasting-pretreated myocardium or cardiomyocytes was preserved during acute extreme hypoxia as a result of the decrease in ATP consumption after the inhibition of anabolism and the increase in ATP production after the decrease in ROS generation, which resulted from enhanced mitophagy. Concurrently, mTOR knockdown could weaken the glycolysis process and increase the OCR levels especially restored ATP-linked respiration in cardiomyocytes under acute hypoxia. These results confirm that fasting attenuates ROS generation and improves mitochondrial oxygen utilization efficiency under acute hypoxia by inhibiting mTOR activity.

In summary, our study showed for the first time that fasting for 72 h could enhance the body’s adaptability to acute extreme hypoxia. We confirmed that mTOR is the core factor associated with fasting that plays a protective role under acute hypoxia. Through the regulation of downstream anabolism and mitophagy pathways, fasting reduces unnecessary ATP consumption and improves mitochondrial oxygen utilization efficiency to preserve ATP production under acute extreme hypoxia, which enables the body to achieve rapid adaptation to acute extreme hypoxia. According to our research, whether certain drugs targeting mTOR could improve the body’s adaptability to acute extreme hypoxia needs to be further explored. Moreover, as a crucial factor regulating cell growth, mTOR is involved in the synthesis of some important cellular proteins. Knocking out the mTOR or an excessive reduction in mTOR will cause severe damage. Consequently, the appropriate degree of regulation of mTOR under acute hypoxia is also a problem worth considering.

## Supplementary information

Supplementary Information (final version)

## Data Availability

Data are available from the corresponding authors upon request.
