# Supplementary material for: Fasting promotes acute hypoxic adaptation by suppressing mTOR-mediated pathways
Source: Cell Death Dis. 2021 Nov 3;12(11):1045. doi: 10.1038/s41419-021-04351-x (PMC8566556; doi:10.1038/s41419-021-04351-x)
Supplement: Supplementary file 1 — Supplementary Information (final version) [file 41419_2021_4351_MOESM1_ESM.docx]

**Supplemental Information**

**Fasting promotes acute hypoxic adaptation by suppressing mTOR-mediated pathways**

**Ruzhou Zhao, Xingcheng Zhao, Xiaobo Wang, Yanqi Liu, Jie Yang, Shuai Jiang, Xiang Zhou, Bo Jiao, Lin Zhang, Yong Liu, Zhibin Yu**

**Supplemental Figures S1-S5**

**Supplemental Table S1-S3**


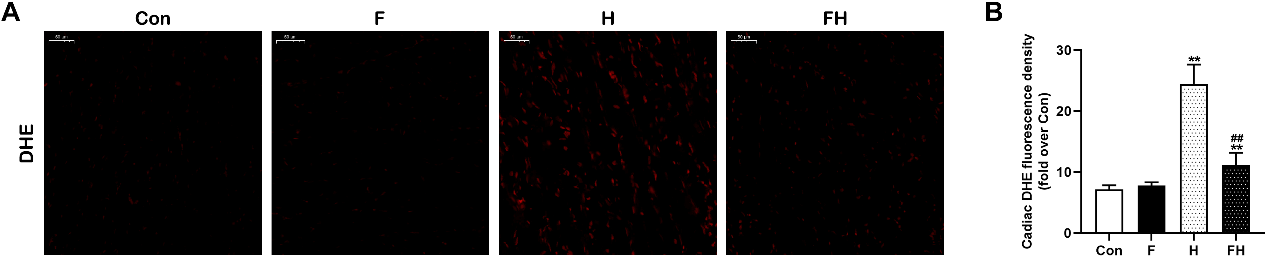


**Figure S1. Fasting pretreatment reduced ROS generation in the myocardium under acute extreme hypoxia**

(A) Representative images of DHE staining in heart sections. Scale bars = 50 μm. (B) Quantitative analysis of DHE fluorescence intensity (fold over Con). The values are presented as the mean ± SEM (n = 5 animals/group). ^**^*P* < 0.01 vs. the Con group. ^##^*P* < 0.01 vs. the H group.


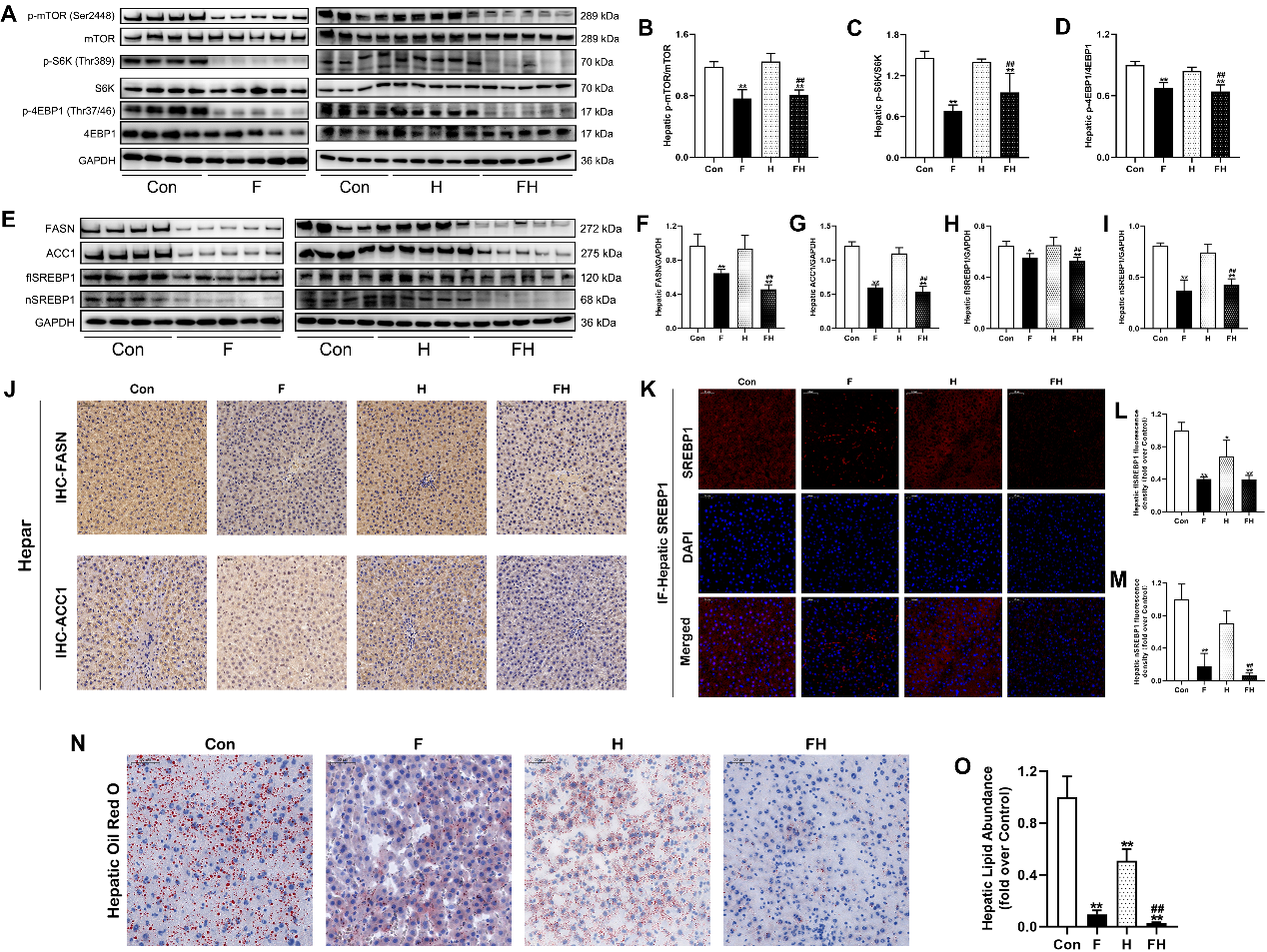


**Figure S2.** **Fasting pretreatment decreased protein synthesis and lipogenesis in hepatocytes during acute extreme hypoxia**

(A-D) Representative western blot bands and quantitative analysis of protein synthesis-related proteins in hepatic tissue.

(E-I) Representative western blot bands and quantitative analysis of lipogenesis-related proteins in hepatic tissue.

(J) Representative immunohistochemical staining of FASN and ACC1 in hepatic tissue. Scale bars = 50 μm.

(K) Representative immunofluorescent images of hepatic tissue stained with SREBP1 (red) and Hoechst 33258 (blue). Scale bars = 50 μm.

(L and M) Quantitative analysis of flSREBP1 (red) and nSREBP1 (violet) fluorescence density in liver sections.

(N and O) Representative Oil Red O staining and quantification of lipids (marked by red puncta) in liver sections. Scale bars = 50 μm.

In (A) and (E), the samples were derived from the same experiment, and gels/blots were processed in parallel. GAPDH was used as a loading control. The values are presented as the mean ± SEM (n = 5 animals/group). ^*^*P* < 0.05 or ^**^*P* < 0.01 vs. the Con group. ^#^*P* < 0.05 or ^##^*P* < 0.01 vs. the H group.


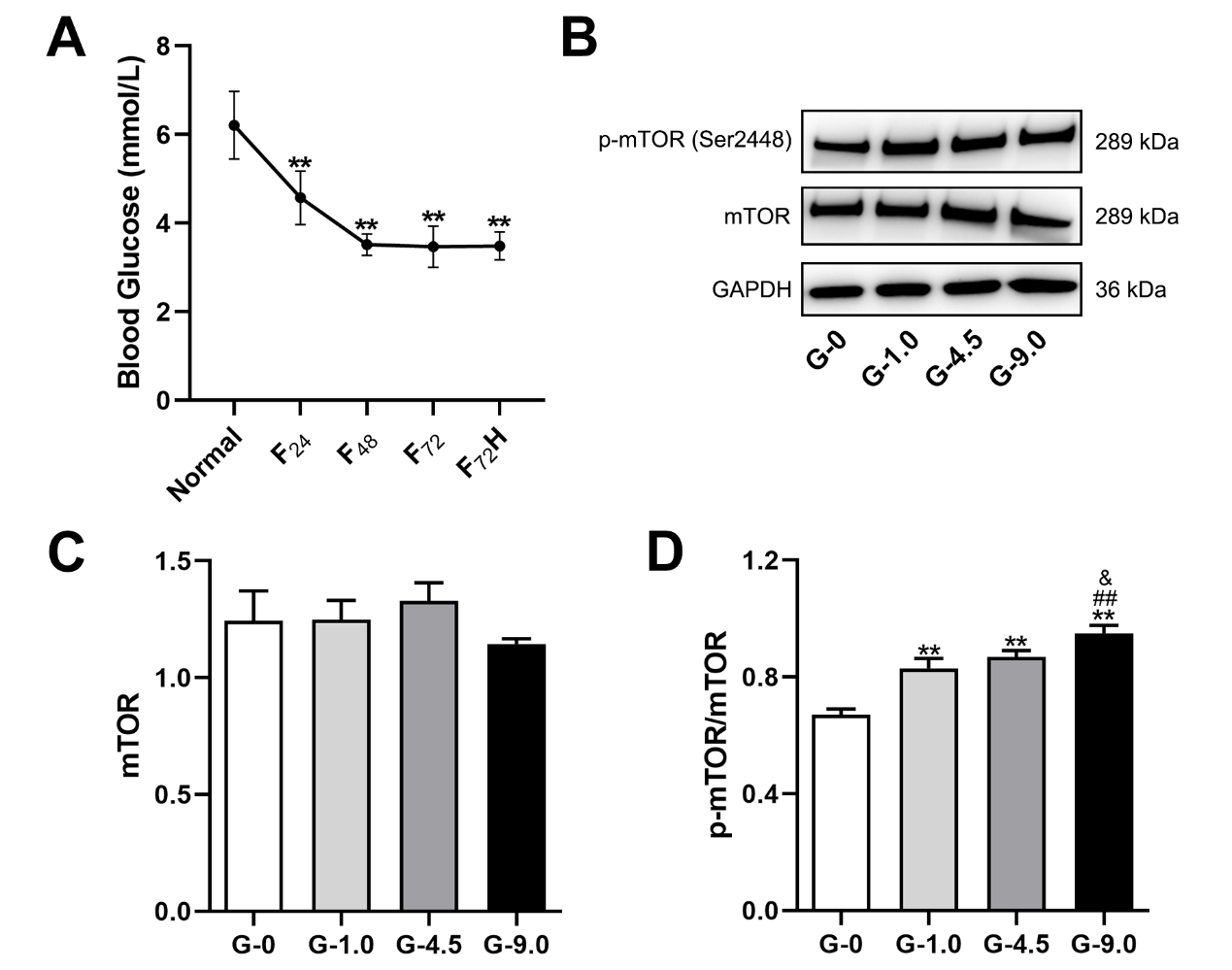


**Figure S3.** **The relationship between mTOR and blood glucose concentration**

(A) Statistical analysis of blood glucose in rats after different fasting times. The values are presented as the mean ± SEM (n = 4-6 animals/group). *^**^P* < 0.01 vs. the Normal group.

(B-D) Representative immunoblots and quantification of p-mTOR/mTOR levels in cardiomyocytes cultured with 0, 1.0, 4.5, and 9.0 g/L glucose. The data represent the mean ± SEM of 3 independent experiments. ^**^*P* < 0.05 vs. the 0 g/L group. ^##^*P* < 0.01 vs. the 1.0 g/L group. ^&^*P* < 0.05 vs. the 4.5 g/L group.


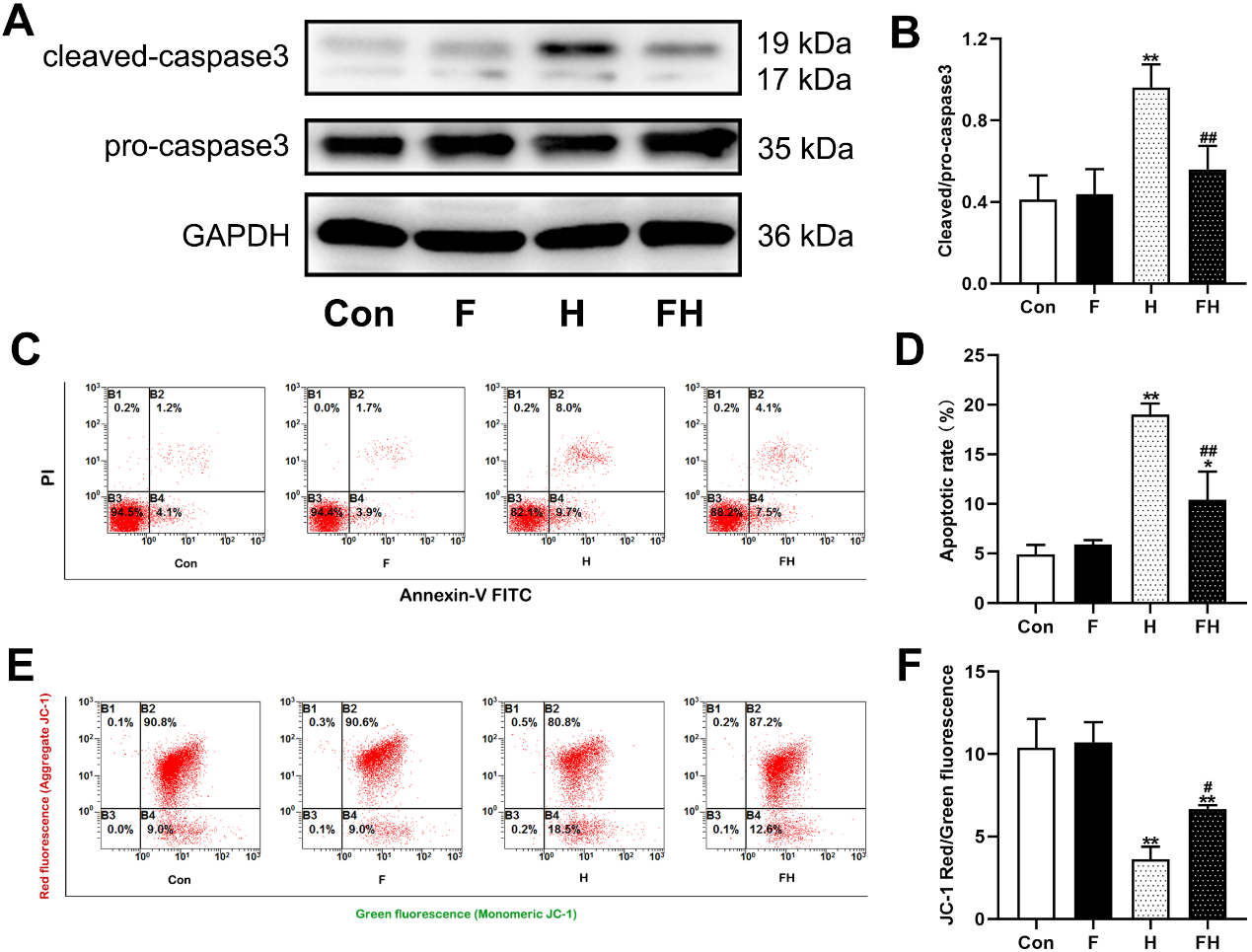


**Figure S4. Simulated fasting preserved MMP and reduced mitochondria-mediated apoptosis in cardiomyocytes during acute hypoxia**

(A and B) Representative blot images and quantification of pro/cleaved caspase 3 expression.

(C) Flow cytometric analysis of apoptosis by Annexin V and PI double-staining in cardiomyocytes. (D) Quantification of total apoptotic cells (bottom right quadrant, viable apoptotic cells; upper right quadrant, nonviable apoptotic cells).

(E and F) Flow cytometric analysis of MMP by JC-1 in cardiomyocytes. Green fluorescence represents low ΔΨm, and red fluorescence represents high ΔΨm. The ΔΨm was quantified by the red/green fluorescence ratio.

The data are presented as the mean ± SEM of three independent experiments. ^*^*P* < 0.05 or ^**^*P* < 0.01 vs. the Con group. ^#^*P* < 0.05 or ^##^*P* < 0.01 vs. the H group.


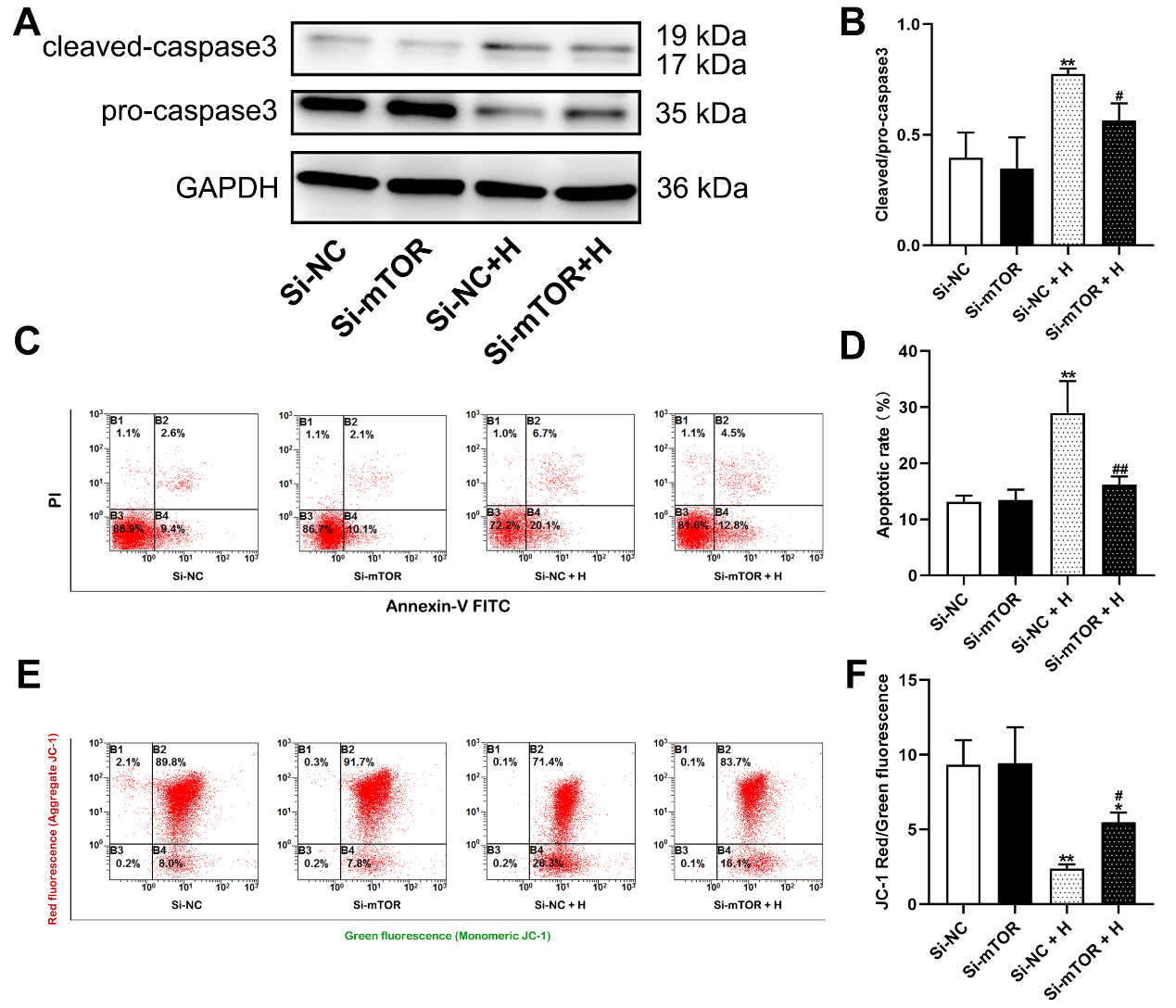


**Figure S5. mTOR gene interference maintained MMP and reduced mitochondria-mediated apoptosis of cardiomyocytes induced by acute hypoxia**

(A and B) Representative blot images and quantification of pro/cleaved caspase 3 expression.

(C and D) Flow cytometric analysis of apoptosis and the quantification of total apoptotic cells among cardiomyocytes.

(E and F) Flow cytometric analysis of MMP by JC-1 in cardiomyocytes and quantification of the red/green fluorescence ratio.

The data are shown as the mean ± SEM of three independent experiments. ^*^*P* < 0.05 or ^**^*P* < 0.01 vs. the Con group. ^#^*P* < 0.05 or ^##^*P* < 0.01 vs. the H group.

**Table S1. Details of primary antibodies**

| **Antibody** | **Manufacturer and Cat No.** | **Application** | **Dilute proportion** |
| --- | --- | --- | --- |
| p-mTOR | Cell Signaling Technology, #5536 | WB | 1:1000 |
| mTOR | Cell Signaling Technology, #2983 | WB | 1:1000 |
| p-S6K | Cell Signaling Technology, #9234 | WB | 1:1000 |
| S6K | Proteintech, #14485-1-AP | WB | 1:2000 |
| p-4EBP1 | Cell Signaling Technology, #2855 | WB | 1:1000 |
| 4EBP1 | Cell Signaling Technology, #9452 | WB | 1:1000 |
| FASN | Proteintech, #10624-2-AP | WB, IHC | 1:800 for WB; 1:200 for IHC |
| ACC1 | Proteintech, #21923-1-AP | WB, IHC | 1:1000 for WB; 1:200 for IHC |
| SREBP1 | Abcam, #28481 | WB, IF | 1:1000 for WB; 1:100 for IF |
| Beclin1 | Proteintech, #11306-1-AP | WB | 1:1000 |
| P62 | Proteintech, #18420-1-AP | WB | 1:1000 |
| LC3 | Abcam, #48394 | WB, IF | 1:1000 for WB; 1:1000 for IF |
| BNIP3 | Abcam, #109362 | WB, IF | 1:1000 for WB; 1:150 for IF |
| Caspase 3 | Cell Signaling Technology, #9662 | WB | 1:1000 |
| GAPDH | Proteintech, #60004-1-Ig | WB | 1:5000 |
| Tom20 | Santa Cruz Biotechnology, #17764 | IF | 1:100 |
| LAMP1 | Affinity, #DF7033 | IF | 1:200 |

**Table S2. Details of second antibodies**

| **Antibody** | **Manufacturer and Cat No.** | **Application** | **Dilute proportion** |
| --- | --- | --- | --- |
| Anti-rabbit, HRP-linked antibody | Cell Signaling Technology, #7074 | WB, IF (double labeling) | 1:5000 |
| Anti-mouse, HRP-linked antibody | Cell Signaling Technology, #7076 | WB | 1:5000 |
| Anti-rabbit, Alexa Fluor 594-linked antibody | Abcam, #150080 | IF | 1:1000 |
| Anti-mouse, Alexa Fluor 488-linked antibody | Abcam, #ab150113 | IF | 1:1000 |

**Table S3. Survival rates of adult SD rats (10 ~ 12 weeks old) with different fasting pretreatment times after exposure to 7620 m for 24 h**

|  | Con | F_24_ | F_48_ | F_72_ |
| --- | --- | --- | --- | --- |
| T | 42 | 18 | 24 | 26 |
| S | 4 | 8 | 17 | 23 |
| R (%) | 9.5 | 44.4 | 70.8 | 88.5 |

Con, normal (no fasting pretreatment) SD rats; F_24_, fasting pretreatment for 24 h; F_48_, fasting pretreatment for 48 h; F_72_, fasting pretreatment for 72 h; T, total number of SD rats in experiments; S, survival number of SD rats after exposure to 7620 m for 24 h; R, survival rate of SD rats after exposure to 7620 m for 24 h.
